# Nifedipine versus atosiban in the treatment of threatened preterm labour (Assessment of Perinatal Outcome after Specific Tocolysis in Early Labour: APOSTEL III-Trial)

**DOI:** 10.1186/1471-2393-14-93

**Published:** 2014-03-03

**Authors:** Elvira OG van Vliet, Ewoud Schuit, Karst Y Heida, Brent C Opmeer, Marjolein Kok, Wilfried Gyselaers, Martina M Porath, Mallory Woiski, Caroline J Bax, Kitty WM Bloemenkamp, Hubertina CJ Scheepers, Yves Jaquemyn, Erik van Beek, Hans JJ Duvekot, Maureen TM Franssen, Bas N Bijvank, Joke H Kok, Arie Franx, Ben Willem J Mol, Martijn A Oudijk

**Affiliations:** 1 Department of Obstetrics and Gynaecology, University Medical Centre Utrecht, Utrecht, the Netherlands; 2 Julius Centre for Health Sciences and Primary Care, University Medical Centre Utrecht, Utrecht, the Netherlands; 3 Clinical Research Unit, Academic Medical Centre, Amsterdam, the Netherlands; 4 Department of Obstetrics and Gynaecology, Academic Medical Centre, Amsterdam, the Netherlands; 5 Department of Obstetrics and Gynaecology, Ziekenhuis Oost-Limburg, Genk, Belgium; 6 Department of Physiology, Hasselt University, Diepenbeek, Belgium; 7 Department of Obstetrics and Gynaecology, Máxima Medical Centre, Veldhoven, the Netherlands; 8 Department of Obstetrics and Gynaecology, Radboud University Nijmegen Medical Centre, Nijmegen, the Netherlands; 9 Department of Obstetrics and Gynaecology, VU Medical Centre, Amsterdam, the Netherlands; 10 Department of Obstetrics and Gynaecology, Leiden University Medical Centre, Leiden, the Netherlands; 11 Department of Obstetrics and Gynaecology, Maastricht University Medical Centre, Maastricht, the Netherlands; 12 Department of Gynecology and Obstaetrics, Antwerp University Hospital, Antwerp, Belgium; 13 Department of Obstetrics and Gynaecology, Sint Antonius Hospital, Nieuwegein, the Netherlands; 14 Department of Obstetrics and Gynaecology, Erasmus Medical Centre, Rotterdam, the Netherlands; 15 Department of Obstetrics, University Medical Centre, University of Groningen, Groningen, the Netherlands; 16 Department of Obstetrics and Gynaecology, Isala Clinics, Zwolle, the Netherlands; 17 Department of Neonatology, Academic Medical Centre, Amsterdam, the Netherlands; 18 The Robinson Institute, School of Paediatrics and Reproductive Health, University of Adelaide, Adelaide, Australia

**Keywords:** Preterm birth, Tocolytics, Nifedipine, Atosiban, Outcome, Drug safety

## Abstract

**Background:**

Preterm birth is the most common cause of neonatal morbidity and mortality. Postponing delivery for 48 hours with tocolytics to allow for maternal steroid administration and antenatal transportation to a centre with neonatal intensive care unit facilities is the standard treatment for women with threatening preterm delivery in most centres. However, there is controversy as to which tocolytic agent is the drug of first choice. Previous trials have focused on tocolytic efficacy and side effects, and are probably underpowered to detect clinically meaningfull differences in neonatal outcome. Thus, the current evidence is inconclusive to support a balanced recommendation for clinical practice. This multicenter randomised clinical trial aims to compare nifedipine and atosiban in terms of neonatal outcome, duration of pregnancy and maternal side effects.

**Methods/Design:**

The Apostel III trial is a nationwide multicenter randomised controlled study. Women with threatened preterm labour (gestational age 25 – 34 weeks) defined as at least 3 contractions per 30 minutes, and 1) a cervical length of ≤ 10 mm or 2) a cervical length of 11-30 mm and a positive Fibronectin test or 3) ruptured membranes will be randomly allocated to treatment with nifedipine or atosiban. Primary outcome is a composite measure of severe neonatal morbidity and mortality. Secondary outcomes will be time to delivery, gestational age at delivery, days on ventilation support, neonatal intensive care (NICU) admittance, length admission in neonatal intensive care, total days in hospital until 3 months corrected age, convulsions, apnoea, asphyxia, proven meningitis, pneumothorax, maternal side effects and costs. Furthermore, an economic evaluation of the treatment will be performed. Analysis will be by intention to treat principle. The power calculation is based on an expected 10% difference in the prevalence of adverse neonatal outcome. This implies that 500 women have to be randomised (two sided test, β 0.2 at alpha 0.05).

**Discussion:**

This trial will provide evidence on the optimal drug of choice in acute tocolysis in threatening preterm labour.

**Trial registration:**

Clinical trial registration: NTR2947, date of registration: June 20th 2011.

## Background

Preterm birth is the most common cause of neonatal morbidity and mortality worldwide [1]. In the USA, the rate of infants born before 37 weeks gestation is 12-13%; while in Europe and other developed countries these rates vary between 5-11% [2,3]. Preterm birth accounts for approximately 75% of all neonatal deaths and 50% of childhood neurological morbidities [4,5] and puts a financial burden on the public health care system [6]. Neurodevelopmental impairments are frequently present in preterm infants, and are associated with gestational age [7]. Neonatal outcome is enhanced by antenatal corticosteroid administration and in-utero transfer to a tertiary care centre [8,9]. To optimize outcome in threatening preterm delivery, postponing delivery for 48 hours with tocolytic agents is common practice in most perinatal centres [10], to allow maximal effect of maternal steroid administration and transportation of the mother to a centre with neonatal intensive care unit (NICU) facilities.

Several types of tocolytic drugs are commonly used as treatment in preterm labour. These include the β adrenoceptor agonist ritodrine hydrochloride, the oxytocin receptor antagonist atosiban and the calcium channel blocking agent nifedipine. Several meta-analyses indicate that tocolytic drugs are superior to placebo or other tocolytics at delaying delivery by 48 hours and 7 days [11,12].

However, controversy exists as to which tocolytic is the drug of first choice. The ideal drug of choice should be efficient in postponing preterm labour, have a favorable safety profile in both mother and fetus, and should reduce neonatal morbidity and mortality at a reasonable cost.

Studies on β adrenoceptor agonists have shown mixed results for postponing delivery compared to placebo [13]. As β adrenoceptor agonists have substantial side effects, use has been largely abandoned from clinical practice. A Cochrane review on calcium channel blockers for inhibiting preterm labour showed that nifedipine significantly reduced delivery within seven days of receiving treatment as compared with any other tocolytic agent (relative risk (RR) 0.76; 95% confidence interval (CI) 0.60 to 0.97) [14]. In addition, as compared to other tocolytics, calcium channel blockers also reduced the frequency of neonatal respiratory distress syndrome (RR 0.63; 95% CI 0.46 to 0.88), necrotising enterocolitis (RR 0.21; 95% CI 0.05 to 0.96), intraventricular haemorrhage (RR 0.59 95% CI 0.36 to 0.98) and neonatal jaundice (RR 0.73; 95% CI 0.57 to 0.93), and the requirement for women to have treatment ceased for adverse drug reaction (RR 0.14; 95% CI 0.05 to 0.36). The Cochrane review on oxytocin receptor antagonists for inhibiting preterm labour failed to demonstrate the superiority of atosiban over β adrenoceptor agonists (RR 0.98; 95% CI 0.68 to 1.41) or placebo (RR 2.5; 95% CI 0.51 to 12.35) in terms of tocolytic efficacy or infant outcomes [15]. On the other hand, atosiban is thought to be completely safe for the mother, whereas nifedipine may cause severe hypotension and fetal death [16]. Such side effects could however not be demonstrated in a previous nationwide study in The Netherlands [17]. Two small studies did not show a difference in effectiveness between nifedipine and atosiban [18,19], however, more side effects were observed with the use of nifedipine, consisting of hypotension, tachycardia headache and vertigo. Neonatal outcome was not reported in one study, the other study did not show a difference.

Recently, a larger study was published, (n = 145) and found fewer failures within 48 hours for atosiban compared with nifedipine [20]. However, nifedipine was associated with a longer postponement of delivery. Neonatal morbidity was comparable between the two groups, although the number of neonatal admissions to the NICU and length of postnatal hospital admission was significantly higher in the atosiban group as compared with the nifedipine group. The main outcome measure of these 3 trials were tocolytic efficacy and tolerability, but the trails may be underpowered to detect clinically meaningfull effects in neonatal outcome. Therefore, the evidence remains inconclusive to support a balanced recommendation for clinical practice as the ultimate goal of tocolysis is not only to postpone delivery, but to improve neonatal outcome. This multicenter randomised clinical trial aims to compare nifedipine and atosiban in terms of neonatal outcome, duration of pregnancy, maternal side effects and costs. The study is conducted within the Dutch Obstetric Consortium, a collaborative effort of obstetric clinics in The Netherlands to perform clinical trials.

## Methods/Design

### Aims

The objective of this study is to compare the effectiveness of the tocolytic agents nifedipine and atosiban in the improvement of neonatal outcome in women with threatened preterm labour with a gestational age between 25 – 34 weeks. Outcome is measured in terms of neonatal mortality and morbidity (chronic lung disease, severe intraventricular haemorrhage, periventricular leucomalacia, culture proven sepsis, necrotizing enterocolitis), gestational age at delivery, maternal side effects and costs.

### Participants/eligibility criteria

We included women with a high risk of preterm birth. Women, aged ≥18 years, with threatened preterm labour and a gestational age between 25 and 34 weeks are eligible for participation in the Apostel III trial. The diagnosis of threatened preterm labour is defined by uterine contractions, at least 3 contractions per 30 minutes, and one of the following: 1) a cervical length of ≤10 mm or 2) a cervical length of 11-30 mm and a positive Fibronectin test or 3) ruptured amniotic membranes. Patients with singleton or twin pregnancies are eligible, independent of the position of the fetus.

Exclusion criteria are presence of a contra-indication for tocolysis (severe vaginal bleeding, signs of fetal distress or intrauterine infection, hypertension or use of anti-hypertensive medication, myocardial infarction (<1 month), unstable angina pectoris), cerclage, > 5 cm cervical dilatation, neonates suspected of chromosomal or structural anomalies and tocolytic treatment for >6 hours prior to arrival in a participating centre.

### Procedures, recruitment, randomization and collection of baseline data

The study will be a nationwide multicentre randomised controlled trial conducted within the Dutch Obstetric Consortium. The Dutch Obstetric Consortium is a research collaboration of obstetric clinics in the Netherlands. All 10 Dutch perinatal centres with NICU facilities will participate in the trial. In addition, 10 large teaching hospitals in the Netherlands and 2 perinatal centres in Belgium will participate in this trial.

Eligible women will be identified by the staff and/or local research coordinator of the participating hospitals. After counselling and reading the patient information form, patients will be asked for written informed consent. We will provide patient information in Dutch and English. After informed consent, baseline demographics, obstetric and medical history of patient will be entered in a web-based database, which will also facilitate randomisation. Randomisation will be performed by a web based computerized program using permuted-block randomisation. Randomisation allocation will be in a 1:1 ratio for Nifedipine or Atosiban, block size will be 4.

As this is a comparison of oral medication and intravenous medication, and as both group are treated with active medication, the study will not be blinded.

### Interventions

Patients are allocated to nifedipine or atosiban for 48 hours. In the nifedipine group, the initial dose will be 2 × 10 mg nifedipine capsules orally in the first hour, followed by 20 mg nifedipine retard per 6 hours for the next 47 hours. In the first hour after starting nifedipine, blood pressure and heart rate will be measured every 15 minutes. If blood pressure remains within the normal limits, treatment will be continued and blood pressure and heart rate will be measured 4 times every 24 hours.

In the atosiban group, a bolus injection of 6.75 mg i.v. in 1 minute, followed by 18 mg/hour for 3 hours, followed by a maintenance dosage of 6 mg/hour for 45 hours.

Antenatal corticosteroids will be administered according to the clinical guideline. Prophylactic treatment with antibiotics is at the decision of the attending physician. When the attending physician considers escape medication, this can be discussed with a perinatologist who will be available for study questions 24 hours per day.

### Outcome measures

#### ***Primary outcome measures***

The primary outcome measure will be a composite of adverse neonatal outcome, including bronchopulmonary dysplasia (BPD), periventricular leucomalacia (PVL) > grade 1, intraventricular haemorrhage > grade 2, necrotising enterocolitis (NEC) > stage 1 [21], culture proven sepsis and in-hospital death.

The diagnosis of BPD will be made according to the international consensus guideline as described by Jobe and Bancalari [22] at time of discharge home or at 36 weeks of corrected gestational age. PVL > grade 1 and intraventricular haemorrhage > grade 2 will be diagnosed by repeated neonatal cranial ultrasound by the neonatologist according to the guidelines on neuro imaging described by de Vries [23] and Ment et al. [24] NEC will be diagnosed according to Bell [21], > stage 1. Culture proven sepsis is diagnosed on the combination of clinical signs and positive blood cultures.

#### ***Secondary outcome measures***

Secondary outcomes will be time to delivery, gestational age at delivery, days on ventilation support, length of admission in neonatal intensive care, convulsions, apnoea, asphyxia, proven meningitis, pneumothorax, total days in hospital until 3 months corrected age. Furthermore we will examine differences in maternal mortality and maternal side effects leading to discontinuation of study medication.

### Follow-up of women and infants

All details of delivery, maternal and neonatal assessments during pregnancy and postpartum are recorded in a web-based Case Report Form (CRF). Details of neonatal admission are also recorded. Long-term follow up of children is dependent on future funding.

### Statistical issues

#### ***Sample size***

The sample size is calculated based on a 10%- reduction of the composite poor neonatal outcome from 25% in the atosiban arm to 15% in the nifedipine arm. With a beta of 0.2 and alpha of 0.05 we have to randomize 500 patients (250 in each arm).

#### ***Data analysis***

Data will be analyzed according to the intention to treat principle. The main outcome variable, ‘adverse neonatal outcome’, will be assessed by calculating rates in the two groups, relative risks and 95% confidence intervals as well as numbers needed to treat. To evaluate the potential of each of the strategies, we will also perform a per protocol analysis, taking into account only those women that were treated according to protocol. Time to delivery will be evaluated by Cox proportional hazards regression and Kaplan-Meier estimates, with account for differing durations of gestation at entry, and will be tested with the Log rank test. The other secondary outcome measures will be approached similarly to the primary outcome measure.

Furthermore, we plan to separately report on the treatment effect in the following subgroups: 1) PPROM versus intact membranes 2) GA < 30 weeks versus > 30 weeks, 3) fibronectin positive women only, 4) women with a cervix length < 10 mm, 5) multiple pregnancies, and 6) women with a history of preterm birth.

#### ***Interim analysis***

An interim analysis is planned after the follow up data of the first 150 women that have been included is obtained. The interim analysis will be performed by an independent person and results will be reported to a data safety and monitoring committee (DSMC) for safety and relevance. As an indication, the trial will be stopped if there is a significant difference in the primary outcome, i.e. a poor neonatal outcome, at p < .005 (2-sided). However, the DSMC is free to make its own judgment.

### Data safety monitoring committee

Serious Adverse Events (SAEs) and Suspected Unexpected Serious Adverse Reactions (SUSARs) will be reported to a Data Safety Monitoring Committee (DSMC). The DSMC can decide to perform an extra interim analysis and, if indicated, terminate the trial prematurely.

### Economic evaluation

We plan an economic evaluation of the costs and health effects of nifedipine and atosiban. The economic evaluation will be set up as a cost-effectiveness analysis (CEA) in which we will calculate the cost per prevented case of poor neonatal outcome. To evaluate cost-effectiveness within a long term horizon, downstream costs associated with poor neonatal outcome are estimated, and included in the analysis as a cost-to-benefit ratio. In a cost-utility analysis, with QALYs calculated from average life expectancy and utilities for severe neonatal morbidity, the incremental cost-effectiveness will be expressed as costs per QALY gained. In sensitivity analyses, the impact of parameter uncertainty and stochastic uncertainty is assessed, and the results are visualized in cost-effectiveness planes and cost-effectiveness acceptability curves.

In our economic analysis, we distinguish three cost stages (antenatal stage, delivery/childbirth stage and postnatal stage), and three cost categories: 1) direct medical costs i.e. all costs in the health care sector 2) direct non-medical costs i.e. costs outside the health care sector that are affected by health status or health care, and 3) indirect costs of the pregnant woman and her partner, for example costs of sick leave. For each stage and cost category, costs are measured as the volumes of resources used multiplied with appropriate valuations based on national reference prices, cost-per-unit estimates, or reimbursement fees.

Volumes of health care resource use are measured alongside the clinical study as part of the CRF as well as with questionnaires. Questionnaires will be based on the iMTA Medical Consumption Questionnaire (MCQ) and the Productivity Costs Questionnaire (PCQ) to collect data regarding health care consumption (e.g. number of GP contacts or outpatient visits, hospital admissions, and drug use), travel and time costs and productivity loss during follow-up at 6-month intervals. These questionnaires will be adapted to include only resources relevant to this study, and to document absence from paid work by the partners.

For an evaluation from a societal perspective, valuations of direct medical resources are estimated comprising ‘true economic’ costs, i.e. including shares of fixed costs and hospital overheads. Dutch reference prices are used where available. Otherwise, costs per unit are estimated for at least one teaching and one non-teaching hospital. Calculations based on reimbursement fees is added to our analysis to represent the payers perspective.

Indirect costs are quantified but remain unvalued. Study-specific costs are excluded from analysis.

### Ethical considerations

This study has been approved by the ethics committee of the Academic Medical Centre Amsterdam (Reference number MEC AMC 09/258) and by the boards of management of all participating hospitals. This trial is registered in the Dutch Trial Register, NTR 2947, http://www.trialregister.nl, date of registration: June 20^th^ 2011.

## Discussion

Preterm birth is an important cause of neonatal morbidity and mortality. Outcome of preterm infants can be improved and health care consumption and costs reduced by postponing delivery for 48 hours with tocolytic agents to allow maximal effect of maternal steroid administration and transportation of the mother to a centre with NICU facilities. The optimal type of tocolytic drug should improve neonatal outcome, be effective in delaying delivery, and safe for both mother and fetus. This trial will provide evidence on these subjects on the tocolytic drugs nifedipine and atosiban.

## Abbreviations

NICU: Neonatal intensive care unit; CRF: Case report form; BPD: Bronchopulmonary dysplasia; PVL: Periventricular leucomalacia; NEC: Necrotising enterocolitis; SAE: Serious adverse events; SUSAR: Suspected unexpected serious adverse reactions; DSMC: Data safety and monitoring committee.

## Competing interests

The authors declare that they have no competing interests.

## Authors’ contributions

MO, BWM, BO and JK were involved in conception and design of the study. MO, BWM and EvV drafted the manuscript. All authors mentioned in the manuscript are member of the Apostel-III study group or collaborators. They are local investigators at the participating centers, and participated in the design of the study during several meetings. All authors edited the manuscript and read and approved the final draft of the manuscript.

## Pre-publication history

The pre-publication history for this paper can be accessed here:

http://www.biomedcentral.com/1471-2393/14/93/prepub
